# Optimal MRI sequences for ^68^Ga-PSMA-11 PET/MRI in evaluation of biochemically recurrent prostate cancer

**DOI:** 10.1186/s13550-017-0327-7

**Published:** 2017-09-19

**Authors:** Spencer T. Lake, Kirsten L. Greene, Antonio C. Westphalen, Spencer C. Behr, Ronald Zagoria, Eric J. Small, Peter R. Carroll, Thomas A. Hope

**Affiliations:** 1 Department of Radiology and Biomedical Imaging, University of California, 505 Parnassus Avenue – 0628, San Francisco, CA 94143-0628 USA; 2 Department of Urology, University of California, 1825 4th Street, 4th floor, UCSF Ron Conway Family Gateway Medical Building, San Francisco, CA 94158 USA; 3 UCSF Helen Diller Family Comprehensive Cancer Center, University of California, San Francisco, CA USA; 4 Division of Hematology/Oncology, Department of Medicine, University of California, 505 Parnassus Avenue, Box 1711, San Francisco, CA 94143-1711 USA; 5 Department of Radiology, San Francisco VA Medical Center, 4150 Clement Street, San Francisco, 94121 CA USA

**Keywords:** Prostate cancer, Prostate-specific membrane antigen, PSMA, PET/MRI, Biochemical recurrence

## Abstract

**Background:**

PET/MRI can be used for the detection of disease in biochemical recurrence (BCR) patients imaged with ^68^Ga-PSMA-11 PET. This study was designed to determine the optimal MRI sequences to localize positive findings on ^68^Ga-PSMA-11 PET of patients with BCR after definitive therapy. Fifty-five consecutive prostate cancer patients with BCR imaged with ^68^Ga-PSMA-11 3.0T PET/MRI were retrospectively analyzed. Mean PSA was 7.9 ± 12.9 ng/ml, and mean PSA doubling time was 7.1 ± 6.6 months. Detection rates of anatomic correlates for prostate-specific membrane antigen (PSMA)-positive foci were evaluated on small field of view (FOV) T2, T1 post-contrast, and diffusion-weighted images. For prostate bed recurrences, the detection rate of dynamic contrast-enhanced (DCE) imaging for PSMA-positive foci was evaluated. Finally, the detection sensitivity for PSMA-avid foci on 3- and 8-min PET acquisitions was compared.

**Results:**

PSMA-positive foci were detected in 89.1% (49/55) of patients evaluated. Small FOV T2 performed best for lymph nodes and detected correlates for all PSMA-avid lymph nodes. DCE imaging performed the best for suspected prostate bed recurrence, detecting correlates for 87.5% (14/16) of PSMA-positive prostate bed foci. The 8-min PET acquisition performed better than the 3-min acquisition for lymph nodes smaller than 1 cm, detecting 100% (57/57) of lymph nodes less than 1 cm, compared to 78.9% (45/57) for the 3-min acquisition.

**Conclusion:**

PSMA PET/MRI performed well for the detection of sites of suspected recurrent disease in patients with BCR. Of the MRI sequences obtained for localization, small FOV T2 images detected the greatest proportion of PSMA-positive abdominopelvic lymph nodes and DCE imaging detected the greatest proportion of PSMA-positive prostate bed foci. The 8-min PET acquisition was superior to the 3 min acquisition for detection of small lymph nodes.

## Background

After initial definitive treatment of prostate cancer with radical prostatectomy, brachytherapy, or external beam radiation, prostate-specific antigen (PSA) may rise due to local, regional, or systemic recurrence prior to the development of clinical symptoms. This rise in pre-clinical PSA is termed biochemical recurrence (BCR). After prostatectomy, BCR is defined by the American Urological Association as a PSA value of 0.2 ng/mL or higher on two separate tests [1]. In patients treated with radiation therapy, including external beam radiation and brachytherapy, the RTOG-ASTRO Phoenix Consensus Conference defined BCR as a rise in PSA by 2.0 ng/mL above the PSA nadir [2]. Patients with BCR may be assessed with a combination of technetium-99m bone scintigraphy and periodic CT scans [3]. However, CT scans do not accurately evaluate lymph nodes, especially those smaller than 5 mm [4], and bone scintigraphy has limited sensitivity and specificity for the detection of osseous metastases, due to radiotracer uptake by non-metastatic lesions, most commonly degenerative diseases.

Prostate-specific membrane antigen (PSMA) is a membrane protein that is overexpressed in prostate cancer cells in comparison to benign prostate cells and increases in advanced stage and androgen-independent prostate cancer [5]. Molecular imaging targeted to PSMA has been shown to have a high detection rate of lesions suspicious for local recurrence and metastatic disease [6, 7]. In two intra-patient comparisons, for example, ^68^Ga-PSMA-11 PET had a higher sensitivity than fluorocholine PET, particularly in patients with a PSA less than 2.0 ng/mL [6, 8].

While PSMA PET is highly sensitive for suspected sites of prostate cancer recurrence, it does not provide precise localization of the PSMA-avid foci. Therefore, PET images need to be co-registered with images from an additional modality for localization, and this has typically been done with CT scans because of availability, ease of acquisition, and high spatial resolution. However, pairing PSMA PET with MRI, which has superior soft tissue resolution than CT, has the potential to provide additional valuable information for the evaluation of BCR patients.

Prior studies that investigated the use of PET/MRI in prostate cancer have focused on patients prior to treatment, given the ability of multiparametric MRI to evaluate primary tumor [9–11]. But combining the molecular, anatomical, and functional imaging data of PET and MRI may also allow for precise localization of disease in patients with BCR. Accordingly, we retrospectively evaluated the optimal MRI sequences to localize positive findings on ^68^Ga-PSMA-11 PET of patients with BCR after prostatectomy or radiation therapy.

## Methods

This study was approved by the institutional review board, and written informed consent was obtained from all patients. This study was performed under an Investigational New Drug approval from the Food and Drug Administration as part of a trial prospectively evaluating the accuracy of ^68^Ga-PSMA-11 for the detection of prostate cancer (NCT02611882). Inclusion in the trial required a PSA doubling time of less than 12 months, and these patients have been reported as part of a change in management analysis [12]. Fifty-five consecutive patients who underwent ^68^Ga-PSMA-11 PET/MRI for BCR from March 2016 to September 2016 were evaluated. All of the patients were previously reported in a study evaluating how ^68^Ga-PSMA-11 PET changed management in patients with BCR [12]. This prior study surveyed the referring clinicians to determine the effect that the imaging results would have on patient management but did not compare the detection rate of different MRI and PET sequences for suspicious lesions, as was done in this study.

### Imaging protocol

Patients were injected with 201.5 ± 52.9 MBq (5.4 ± 1.4 mCi) of ^68^Ga-PSMA-11. All but two patients received furosemide concurrent with the radiotracer injection to prevent scatter artifact from bladder and kidney activity. Images were acquired on a simultaneous 3.0T time-of-flight PET/MRI (Signa, GE Healthcare), and imaging was performed 65 ± 11 min after injection. Two PET acquisitions were obtained: the first PET acquisition included two bed positions covering the abdomen and pelvis with 8 min of PET acquisition at each bed position starting with the pelvis bed position. The second PET acquisition was a whole-body PET acquisition from the mid-thighs to the vertex obtained for 3 min at each of the six bed positions. The following MRI sequences were obtained at the two abdominopelvic bed positions:

1. Small field of view T2: fast spin echo, flip angle = 125**°**, slice thickness = 4.5 mm, number of slices per acquisition = 27, TE/TR = 129/7567, NEX = 1.5, field of view (FOV) = 220 × 220 mm, acquisition matrix 448 × 256
2. Diffusion-weighted images (DWI): axial echo planar DWI, *b* values = 50 and 500, slice thickness = 6 mm, TE/TR = 54/14117, NEX = 1, matrix = 128 × 100
3. Dynamic contrast-enhanced (DCE) imaging with Differential Subsampling with Cartesian Ordering (DISCO) [13]: obtained at the pelvis bed position, slice thickness = 2 mm, flip angle = 15**°**, matrix = 512 × 512, TE1/TE2/TR = 2.0/4.1/5.6 msec, NEX = 0.7, parallel imaging acceleration factors of 2 (phase direction) × 2.5 (slice direction). Following a single dose (0.1 mmol/kg) of gadobutrol (Gadavist, Bayer Healthcare, Berlin, Germany), 17 phases were acquired sequentially with an 11-s temporal resolution. Three patients did not undergo DCE imaging
4. Post-contrast T1-weighted images (LAVA-FLEX): axial 3D spoiled gradient echo sequence using two-point Dixon fat saturation, slice thickness = 3 mm, flip angle = 12**°**, matrix size = 316 × 256, TE1/TE2/TR = 2.0/4.1/5.6, NEX = 0.7.

A whole-body MRI was also obtained at six bed positions while completing the whole-body PET scan:

1. Axial MR attenuation correction: slice thickness = 5.2 mm, flip angle = 5°, matrix size = 256 × 128, TE1/TE2/TR = 1.2/2.3/5.2, NEX = 0.7. The data were converted to an attenuation map as previously described [14, 15]
2. Axial and coronal single-shot fast-spin echo (SSFSE): TE/TR = 100/576, acquisition matrix 320 × 224, axial slice thickness = 5 mm, coronal slice thickness = 6.5 mm. A variable refocusing flip angle was used as previously described [16]
3. Axial T1 post-contrast images (LAVA-FLEX): parameters identical to the abdomen and pelvis acquisition

### Image analysis

For each study, up to five PSMA-positive foci were identified on PSMA PET and grouped into prostate bed, pelvic lymph nodes, retroperitoneal lymph nodes, thoracic lymph nodes, cervical lymph nodes, visceral lesions, and osseous lesions, which were further subcategorized as spine/pelvis, rib, or other osseous lesions. When more than five PSMA-positive foci were present, representative lesions were chosen for analysis, with no more than two lesions in each of the possible categories when possible. A diagnostic radiologist recorded the presence or absence of an identifiable correlate for each radiotracer-avid focus. For disease in the abdomen and pelvis, the small FOV T2, DWI, and T1-post-contrast images were analyzed to determine the detection rates of foci with PSMA uptake. For each lesion, long- and short-axis measurements were obtained on the sequence that best identified it. For lesions above the diaphragm, the presence of an identifiable correlate for each radiotracer-avid focus was evaluated utilizing the whole-body SSFSE and T1-post-contrast images, and for each lesion, long- and short-axis measurements were obtained on the sequence that best identified it. PET-avid foci were recorded on both the 8-min abdominopelvic acquisition (when applicable) and on the 3-min whole-body PET acquisition. A board-certified, fellowship-trained, abdominal radiologist with 4 years of experience with prostate MRI reviewed all MRI sequences to detect recurrences in the prostate bed. Lesions detected in the prostate bed on MRI without PSMA uptake were considered positive. These were recorded separately and not included in the calculated detection rate of MRI for PSMA-positive lesions, given the absence of PSMA uptake.

### Statistical analysis

A chi-square test was used to determine statistical significance of differences between proportions of lesions detected by difference MRI sequences. A level of significance of *p* < 0.05 was used. The 95% confidence intervals were also calculated. The statistical analysis was conducted using MedCalc for chi-square tests and www.sample-size.net for confidence intervals.

## Results

Table 1 summarizes the demographics, and baseline and post-treatment clinical characteristics of the study patient population. Sixteen men underwent primary radical prostatectomy (16/55, 29.1%), 18, primary radiation therapy—external beam radiation and/or brachytherapy (18/55, 32.7%), and 21, prostatectomy and radiation therapy (21/55, 38.2%). The mean PSA at time of PET/MRI was 7.9 ± 12.9 ng/ml, and the mean PSA doubling time was 7.1 ± 6.6 months.

**Table 1:** Patient data

Number of patients	55
Age (years)	
Mean (SD)	68.3 (6.9)
Range	48–83
Gleason score	
3 + 3	9
3 + 4	16
4 + 3	12
4 + 4	6
5 + 3	1
4 + 5	9
5 + 4	1
Unknown	1
PSA at diagnosis (ng/mL)	
Mean (SD)	11.2 (12.5)
Range	4.0–88.0
Post-treatment PSA nadir (ng/mL)	
Mean (SD)	0.5 (1.1)
Range	0–5.1
Post-treatment PSA (ng/mL)	
Mean (SD)	7.9 (12.9)
Range	0.2–84.0
PSA doubling time (months)	
Mean (SD)	7.1 (6.6)
Post-treatment PSA grouping (n)	
0–1 ng/mL	8 (14.5%)
1–2 ng/mL	10 (18.2%)
> 2 ng/mL	37 (67.3%)
Treatment (*n*)	
Prostatectomy	16 (29.1%)
Radiation	18 (32.7%)
Prostatectomy and radiation	21 (38.2%)

### PSMA PET MRI

PSMA-avid foci were detected in 49 patients (49/55, 89.1%): 75.0% (6/8) of patients with a PSA between 0 and 1 ng/mL, 80.0% (8/10), with PSA between 1 and 2 ng/mL, and 94.6% (35/37), with a PSA greater than 2 ng/mL. Nineteen patients (19/55, 34.5%) had positive PSMA foci suspicious for local recurrence in the prostatic bed. Twenty-nine patients (29/55, 52.7%) had pelvic or retroperitoneal lymph nodes that were positive on PSMA PET. Fourteen patients (14/55, 25.5%) had suspected osseous metastases on PSMA PET. No patients had suspicious visceral uptake. Table 2 details the sites of suspected recurrent disease on PSMA PET.

**Table 2:** Sites of suspected recurrence on PSMA

Sites of recurrence	Patients	Post RP (*n* = 16)	Post RT (*n* = 18)	Post RP and RT (*n* = 21)
Prostate bed	21	4	15	2
Pelvic lymph nodes	28	8	8	12
Retroperitoneal lymph nodes	13	4	3	6
Thoracic lymph nodes	1	0	1	0
Cervical lymph nodes	3	0	1	2
Bone (spine/pelvis)	7	2	0	5
Bone (ribs)	6	0	3	3
Bone (other)	7	1	2	4
Visceral metastases	0	0	0	0
None	6	4	0	2

*RP* radical prostatectomy, *RT* radiation therapy

### Nodal disease

**Fig. 1:**
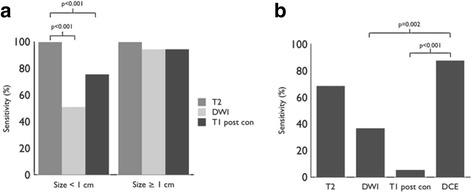
Detection rate for abdominopelvic lymph nodes by size and MRI sequence (**a**). Small FOV T2, DWI, and T1 post-contrast images identify nearly all PSMA-positive lymph nodes greater than 1 cm. Small FOV T2 is more sensitive than DWI or T1 post-contrast images for lymph nodes less than 1 cm (*p* < 0.001). Detection rate for prostate bed recurrence by MRI sequence (**b**). Dynamic contrast-enhanced imaging (DCE) is more sensitive than DWI or single-phase T1 post-contrast images for suspected PSMA-avid prostate bed recurrence (*p* = 0.002 and *p* < 0.001, respectively). The difference in sensitivity between DCE and T2 was not statistically significant (*p* > 0.05)

**Fig. 2:**
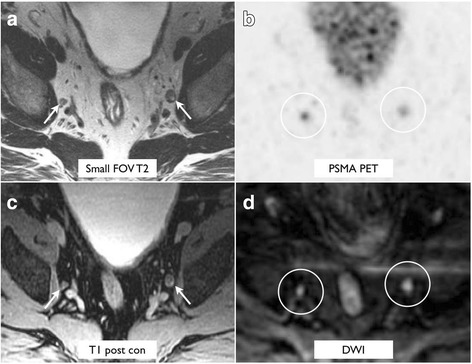
A 67-year-old man with biochemical recurrence (PSA of 8.0), who had bilateral pelvic side wall lymph nodes measuring 0.7 and 1.0 cm seen on T2-weighted imaging (**a**), PSMA PET (**b**), post-gadolinium T1-weighted imaging (**c**), and DWI (**d**)

**Fig. 3:**
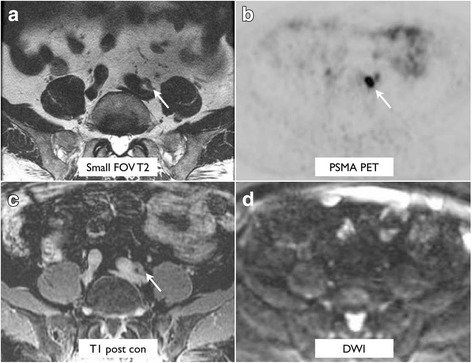
A 70-year-old man with biochemical recurrence (PSA of 10.5), who had a left common iliac lymph node measuring 1.3 × 0.6 cm seen on T2-weighted imaging (**a**), PSMA PET (**b**), and post-gadolinium T1-weighted imaging (**c**). The lymph node was not seen on diffusion-weighted imaging (**d**)

T2-weighted imaging performed the best for localization of small PSMA-positive lymph nodes (Tables 3 and 4 and Fig. 1a). The higher localization rate of T2-weighted imaging for lymph nodes less than 1 cm in comparison to T1 post-contrast and DWI was statistically significant (*p* < 0.001). Example lesions are demonstrated in Figs. 2 and 3.

**Table 3:** Correlate detection for PSMA-avid foci by sequence in the abdomen or pelvis

Location	Total lesions	Small FOV T2	DWI	T1 post-contrast	DCE
Pelvic lymph nodes					
> 1 cm	15	15	14	14	
< 1 cm	39	39	19	28	
Retroperitoneal lymph nodes					
> 1 cm	3	3	3	3	
< 1 cm	18	18	10	15	
Prostate bed	19	13	7	1	14

^a^Three patients who had PSMA-positive foci in the prostate bed did not undergo DCE imaging

**Table 4:** Detection rates by MRI sequence for abdominopelvic lymph node correlates to PSMA-positive foci

Sequence	Percent detection by sequence (95% CI)		
	Nodes < 1 cm	Nodes > 1 cm	Prostate bed
Small FOV T2	100 (93.7–100)	100 (81.5–100)	68.4 (43.4–87.4)
DWI	50.9 (37.3–64.4)	94.4 (72.7–100)	36.8 (16.3–61.6)
T1 post-contrast	75.4 (62.2–85.9)	94.4 (72.7–100)	5.3 (0–26.0)
DCE			87.5 (61.7–98.4)

### Prostate bed lesions

**Fig. 4:**
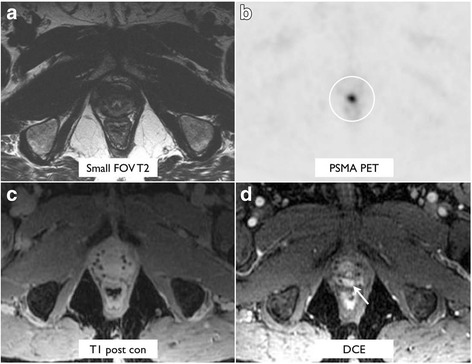
A 62-year-old man with biochemical recurrence (PSA of 3.2), who had suspected PSMA-avid prostate bed recurrence measuring 1.0 × 0.6 cm after initial treatment with brachytherapy, seen on PSMA PET (**b**) and DCE images (**d**). The local recurrence was not seen on T2-weighted imaging (**a**), post-gadolinium T1-weighted imaging (**c**), or DWI (not shown due to artifact from brachytherapy seeds)

DCE MRI performed better than other MRI sequences for localization of correlates for suspected prostate bed recurrence on PSMA PET (Tables 3 and 4 and Fig. 1b). The difference in sensitivity between DCE and DWI or single-phase T1 post-contrast was statistically significant (*p* = 0.002 and *p* > 0.001, respectively). The difference in sensitivity between DCE and T2 was not statistically significant (*p* > 0.05). An example lesion is demonstrated in Fig. 4. Two additional prostate bed lesions were detected on DCE MRI without associated PSMA uptake.

### Osseous lesions

Small FOV T2 and contrast-enhanced T1-weighted images were more sensitive for correlates for PSMA-positive bone foci than SSFSE. Small FOV T2 images identified correlates for 100% (5/5) of these suspected bone lesions less than 1 cm and 100% (4/4) suspected bone lesions greater than 1 cm in the abdomen and pelvis. Contrast-enhanced T1 images identified correlates for 100% (14/14) suspected bone lesions less than 1 cm and 88.9% (8/9) suspected bone lesions greater than 1 cm in the whole body. SSFSE images identified correlates for 53.8% (7/13) suspected bone lesions less than 1 cm and 62.5% (5/8) suspected bone lesions greater than 1 cm in the whole body, with two lesions not included in the SSFSE field of view.

### Lesion detection on long and short PET acquisitions

**Fig. 5:**
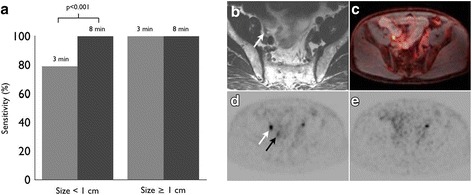
Detection rate for abdominopelvic lymph nodes by size on 3- and 8-min PET acquisitions (**a**). The 8-min PET is more sensitive for lymph nodes smaller than 1 cm (*p* < 0.001). The 3- and 8-min PET detected all lymph nodes larger than 1 cm. Example, 0.7-cm right external iliac lymph node visible on T2-weighted images (**b**), fused PET/MR images (**c**), and on 8-min PET acquisition (**d**), but not seen on 3-min PET acquisition (**e**). The right ureter is designated with a black arrow

The 8-min PET acquisition outperformed the 3-min acquisition for the detection of suspected nodal metastases less than 1 cm (Fig. 5). The 8-min acquisition detected 100% (75/75) of abdominopelvic lymph nodes. The 3-min acquisition detected 84.0% (63/75) of abdominopelvic lymph nodes, including 100% (18/18) lymph nodes greater than 1 cm and 78.9% (45/57) lymph nodes less than 1 cm (*p* < 0.001 for lymph nodes less than 1 cm).

## Discussion

In this study, we demonstrated that T2-weighted imaging is the most effective MRI sequence for identifying anatomic correlates to ^68^Ga-PSMA-11 PET-avid abdominopelvic lymph nodes, and DCE is most effective for identifying PSMA-avid foci in the prostatic bed. Also, longer PET acquisition times increased the detection sensitivity for subcentimeter PSMA-avid foci.

Early and accurate detection of sites of disease in patients with BCR allows for patient-specific treatments, for example, targeted external beam radiation to the prostate bed or sites of oligometastatic disease, or systemic therapy for more widespread disease [17]. It is important, therefore, to be able to precisely localize sites of persistent disease. ^68^Ga-PSMA-11 PET/MRI may be especially important in patients with low serum PSA levels, who may present with subcentimeter nodes. The high incidence of PSMA-positive lymph nodes less than 1 cm in size indicates that MRI alone would not be as sensitive for detection of abnormal nodes.

The high sensitivity of PSMA in the study patient population (89.1%) mirrored that demonstrated in prior studies [6, 7]. The use of PET/MRI rather than PET/CT has the potential to detect even more lesions, especially in the prostate bed, where multiparametric MRI has been shown to be highly sensitive for detection of suspected local recurrence, including for tumor recurrence that is not evident on PET [18]. In particular, our results illustrate the utility of DCE MRI to detect PSMA-avid foci in the prostatic bed, with a localization rate of 87.5%. DCE MRI has previously been shown to have a high sensitivity: Haider et al. demonstrated a sensitivity of 72% of DCE for recurrence after external beam radiotherapy, and Rischke et al. demonstrated a sensitivity of 67% of DCE for recurrence after radical prostatectomy [19, 20], which is also reflected in a meta-analysis by Wu et al. [21]. The limitations that were observed in sensitivity of T2-weighted imaging and DWI are likely due to postoperative and radiation changes in the prostate as well as artifact from brachytherapy seeds [22–24]. Prior studies have suggested moderate to high accuracy of DWI for the detection of recurrent prostate cancer after radiation therapy [25, 26]. These results suggest that DCE should be an integral part of a PET/MRI protocol that aims to detect recurrent tumor in a background of treatment changes.

Previous work has shown that prostate MRI can be more sensitive than PSMA PET for suspected local recurrence, especially for lesions in close proximity to the bladder, due to severe scatter artifact associated with activities in the bladder [18]. This is a potential benefit of pairing PSMA PET with MRI instead of with CT. In our study, two additional lesions were detected, which were identified only on DCE MRI. Scatter did not appear to limit identification of PSMA-avid lesions on the PET sequences in our study. It is possible that time-of-flight reconstructions or furosemide administration resulted in lower artifact compared to what has been reported previously using non-time-of-flight PET/MRI systems [18]. With newer PSMA-targeted imaging agents that have more prominent biliary excretion, scatter artifact will likely become less of an issue [27, 28].

Although multiparametric MRI (mpMRI) alone or in conjunction with PSMA PET is highly sensitive for the detection of prostatic bed recurrence [18–21, 29], disease is frequently outside the prostatic bed. Thirty of 49 patients with PSMA-positive disease had no evidence of recurrent cancer in the prostatic bed, but had PSMA uptake elsewhere. Since sites of suspected recurrence may be found in many regions outside of prostatic bed, the combination of mpMRI of the prostate, whole-body MRI, and PSMA PET is promising for a comprehensive evaluation of patients with BCR. Although nearly all PSMA-avid lesions had an MRI correlate, these lesions would not have been interpreted as metastatic lesions without PSMA PET. Of note, we did not evaluate osseous lesions that were not PSMA avid, nor was our protocol optimized to characterize osseous lesions in the absence of PSMA avidity.

One of the unexpected benefits of simultaneous PSMA PET/MRI in this study was the added value of the 8-min PET acquisition compared to the 3-min acquisition for the detection of uptake within small lymph nodes. This is longer than the recommendation in the EANM/SNMMI imaging guidelines [30]. The increased acquisition time was initially implemented to take advantage of the time used to perform the simultaneous mpMRI. The 25 cm *z*-axis length of the detector in both available simultaneous PET/MRIs allows for doubling the field of view coverage compared to available PET/CTs, thereby allowing for the doubling of the PET acquisition time without changing the length of the study. Though increased uptake time is associated with increased detection sensitivity [31], the 8-min acquisition was performed before the 3-min acquisition, so this likely did not bias the results towards the longer acquisition. In future implementations, the 8-min acquisition will be combined into the whole-body protocol, allowing for a complete whole-body PET/MRI to be performed in 30 min.

This study has several limitations. First, this is a retrospective study, and there is no histopathological correlation of disease for the PSMA-positive foci that were detected. The majority of patients were treated according to the findings of their PET/MRI scans without biopsy confirmation. It is possible that there were false-positive PSMA PET-avid foci, although no pathology proven false-positive lesions are known. The goal of this study was to identify which MRI sequences should be used to co-localize suspicious findings, rather than estimating the diagnostic accuracy of PSMA PET/MRI. Additionally, only a small number of limited MRI sequences were obtained for localization of PSMA-positive lesions. It is possible that optimized, dedicated, MRI sequences would have a better diagnostic performance; yet, the goal of the study was not to estimate the diagnostic accuracy of MRI for the detection of PSMA PET-avid foci, but to identify the best sequences to co-localize suspicious findings.

## Conclusions

A PSMA PET/MRI scan obtained for the assessment of men with BCR after treatment of prostate cancer should include small FOV T2 images of the abdomen and pelvis and DCE MRI of the pelvis to improve co-localization of suspicious findings identified on PSMA PET. In addition, a longer 8-min PET acquisition may improve the detection of small foci of a suspected recurrent cancer.

## Abbreviations

BCR: Biochemical recurrence
DCE: Dynamic contrast enhanced
DISCO: Differential Subsampling with Cartesian Ordering
DWI: Diffusion-weighted imaging
FOV: Field of view
LAVA: Liver acquisition with volume acquisition
PSMA: Prostate-specific membrane antigen
